# Capecitabine-induced oral mucosal hyperpigmentation associated with hand-foot syndrome – A literature review^⋆^

**DOI:** 10.1016/j.abd.2022.05.004

**Published:** 2023-02-17

**Authors:** Anna Danielly Almeida do Nascimento, Débora Maria Porto, Aurora Karla de Lacerda Vidal

**Affiliations:** Oncology Center, Hospital Universitário Oswaldo Cruz, Universidade de Pernambuco, Recife, PE, Brazil

**Keywords:** Cancer, Capecitabine, Hand-foot syndrome, Oral mucosa, Pigmentation disorders

## Abstract

**Background:**

Capecitabine (Xeloda®) is a cytotoxic, antimetabolite chemotherapeutic agent. Its most common adverse events are diarrhea, hand-foot syndrome (HFS), hyperbilirubinemia, hyperpigmentation, fatigue, abdominal pain, and other gastrointestinal effects. HFS or palmar-plantar erythrodysesthesia (PPE) is an adverse reaction resulting from therapy with chemotherapeutic agents, classified into three degrees. Hyperpigmentation, as an adverse effect of capecitabine, can occur in different locations and with different patterns. The skin, nails and oral mucosal membrane can be affected.

**Objective:**

The objective of this study was to report and discuss oral hyperpigmentation associated with HFS caused by the use of capecitabine, which is still poorly described in the literature.

**Methodology:**

A literature review was carried out using the online databases PubMed, Scielo, BVS, Lilacs, Medline, BBO and Google Scholar, associating the descriptors “Capecitabine”, “Pigmentation Disorders”, “Oral mucosa”, “Cancer” and “Hand-Foot Syndrome”, which were related and used to exemplify, discuss and report the exposed clinical case.

**Results:**

This case report corroborates the literature regarding the incidence in females and black skin persons like this patient who was affected by HFS when undergoing antineoplastic therapy with capecitabine and presented hyperpigmentation of the hands, feet and oral mucosa. On the oral mucosa, the hyperpigmented spots were diffuse, showing a blackish color and irregular edges. Their pathophysiology remains unknown.

**Study limitations:**

Few articles citing capecitabine-associated pigmentation.

**Conclusions:**

It is hoped that this study may contribute to the identification and correct diagnosis of hyperpigmentation in the oral cavity, as well as call attention to the adverse effects related to capecitabine.

## Introduction

Antineoplastic drugs, widely used in the treatment of cancer, constitute a heterogeneous group of chemical substances capable of inhibiting the growth and/or vital processes of tumor cells, aiming to destroy their disordered multiplication^1^. They can be administered by oral, subcutaneous, intra-arterial, intramuscular, intrathecal, intraperitoneal, and intravesical routes, by topical and intrarectal application, with the intravenous route being the most frequently used. These drugs can be grouped into the following categories: alkylating agents, antimetabolites, platinum compounds, plant alkaloids, antitumor antibiotics, enzymes, hormones, and biological response modifiers^2^, ^3^, ^4^.

Capecitabine (Xeloda®) is a cytotoxic, antimetabolite chemotherapeutic agent that has been approved for use since 1998. It is a widely used orally administered fluoropyrimidine (5′-deoxy-5-fluorouridine) prodrug that is effective and well tolerated in the treatment of a range of cancers, including breast, colon and rectal, gastric, pancreatic, and others^5^, ^6^. Capecitabine (4-pentyloxycarbonyl-5′-deoxy-5-fluorocytidine) was designed to be enzymatically converted into its active form, 5-Fluorouracil (5-FU), which acts as an antimetabolite to slow tumor growth^7^, ^8^.

Because this medication is administered through the oral route, its advantage over 5-FU is the fact that it is easier to administrate, since 5-FU is injectable, with a short half-life, in addition to the need for central venous access, generating greater discomfort for the patient, and risk of complications, such as infection^8^.

Capecitabine is generally well tolerated and has an improved tolerability profile compared to 5-FU, but it has a nonspecific effect and ends up damaging healthy tissue cells as well, causing adverse effects. Its most common dose-limiting adverse events are diarrhea, hand-foot syndrome (HFS), hyperbilirubinemia, hyperpigmentation, fatigue, abdominal pain, and other gastrointestinal effects, such as nausea/vomiting and stomatitis^4^, ^5^, ^9^. Most reactions are reversible with dose reduction or by increasing the intervals between the doses^10^.

HFS was described in association with chemotherapy for the first time by Zuehlke, in 1974. The National Cancer Institute (NCI)^11^ of the United States classifies adverse events of HFS into three grades as follows:

• Grade I: The patient has minimal skin alterations, such as numbness, dysesthesia, paresthesia, edema, erythema and/or discomfort in the hands and/or feet, but there is no pain;
• Grade II: The patient has manifestations of painful erythema, desquamation, fissures and edema in the hands and/or feet, affecting the performance of daily activities;
• Grade III: The patient has severe skin alterations, such as wet desquamation, edema, ulcerations, blistering, with intense pain and limitation of daily, self-care and work activities.

Hyperpigmentation, as an adverse effect of capecitabine use, can occur in different locations and with different patterns. The skin, nails and mucous membranes, such as the oral mucosa, can be affected. They can appear days to months after starting the treatment and most often resolve within months of therapy discontinuation^4^, ^12^. It is classified by the NCI into 2 grades^11^:

• Grade I: Hyperpigmentation affecting less than 10% of the body area, with no psychosocial impact;
• Grade II: Hyperpigmentation affecting more than 10% of the body area, associated with psychosocial impact.

The clinical manifestations of drug-related pigmentations vary, and mostly present as a diffuse, scattered melanosis on the skin and oral mucosal surface, but may cause a single lesion of uniform patch pattern^13^. When induced by drugs, the spots are more blackish in color and exhibit irregular borders, resembling malignant lesions such as melanomas, emphasizing the importance of the differential diagnosis^14^.

It is important that health professionals, such as dentists and physicians, know how to identify the clinical changes in the color of the mucosa caused by the systemic use of antineoplastic drugs, considering the differential diagnosis with several other oral manifestations expressed through color changes.

The aim of this study was to report hyperpigmentation of the oral mucosa associated with HFS due to the use of capecitabine, which is still little described in the literature.

## Method

A literature review was carried out using the online databases PubMed, Scielo, BVS, Lilacs, Medline, BBO and Google Scholar, associating the descriptors "Capecitabine", "Pigmentation Disorders", "Oral Mucosa", "Cancer" and " Hand-Foot Syndrome”, which were related and used to exemplify, discuss and report the exposed clinical case. There were no language restrictions and all types of studies were considered.

The patient described in the case report signed the Free and Informed Consent form, agreeing to the disclosure of her case for academic purposes. This subproject is part of a project approved by the Research Ethics Committee of Universidade de Pernambuco under Counsel n. 3.184.856, carried out at the Oncology Center of Hospital Universitário Oswaldo Cruz, Universidade de Pernambuco – CEON/HUOC/UPE, located in the city of Recife, state of Pernambuco Brazil.

The pictures that illustrate the study were obtained from the Dental Service Collection of the Oncology Center of Hospital Universitário Oswaldo Cruz, Universidade de Pernambuco CEON-ODONTO/HUOC/UPE.

## Results

### Clinical case

A 54-year-old obese black female patient, was diagnosed with triple-negative invasive ductal carcinoma of the right breast, stage T2N0M0 (AP: 176123), in 2018 and was followed at the Oncology Center of Hospital Universitário Oswaldo Cruz, Universidade de Pernambuco, in Recife, state of Pernambuco (CEON/HUOC/UPE). Initially, a mastectomy with axillary dissection and chemotherapy with an AC-T regimen (Doxorubicin, Cyclophosphamide, and Paclitaxel) were performed up to January 2019. In December 2019, partial collapse of the L3 vertebra occurred, and the diagnosis of recurrence with bone metastasis was made, and radiotherapy was indicated for the lumbar spine (3000 cGy) and, in January 2020, chemotherapy with capecitabine and zoledronic acid (Zometa®) was started.

**Figure 1:**
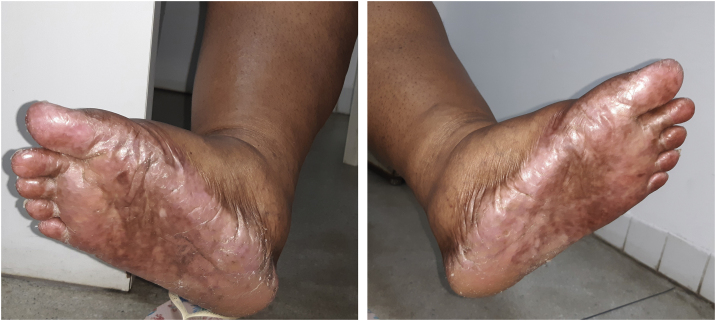
Hyperpigmentation, desquamation and dryness on the soles and inner region of the feet.

The recommended dose for capecitabine monotherapy is 1250 mg/m^2^ twice daily, equivalent to a daily dose of 2500 mg/m^2^. Her body surface was equivalent to approximately 1.5 m^2^, so the prescribed dose was 3000–3500 mg daily for 21 days, followed by seven days without the drug. After two cycles of capecitabine use, the patient started to present HFS, characterized by skin redness, dryness and swelling of the palms and soles, with a tingling or burning sensation, and she started showing hyperpigmentation on the hands, feet (Fig. 1) and oral mucosa. There was no history of similar lesions or adverse reactions to other drugs.

The HFS ranged from grade 1–3 throughout the treatment, with the appearance of bullae on the feet and hands in the most advanced grade, preventing the patient from carrying out daily activities, which required the discontinuation of the medication and topical use of moisturizers with urea, at 10% or 20%, on the hands and feet.

**Figure 2:**
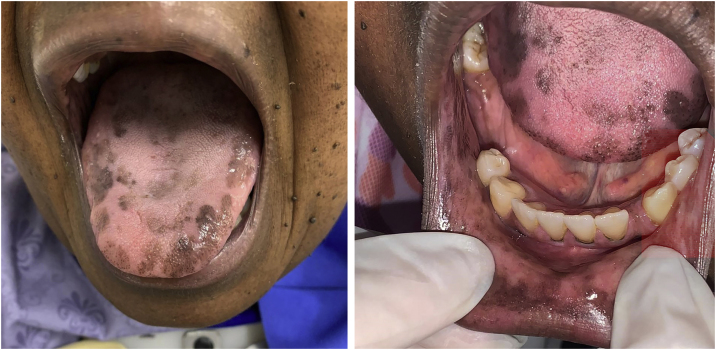
Clinical aspect of hyperpigmented spots on the oral and perioral region, located on the tongue, lip and bottom of the oral vestibule.

**Figure 3:**
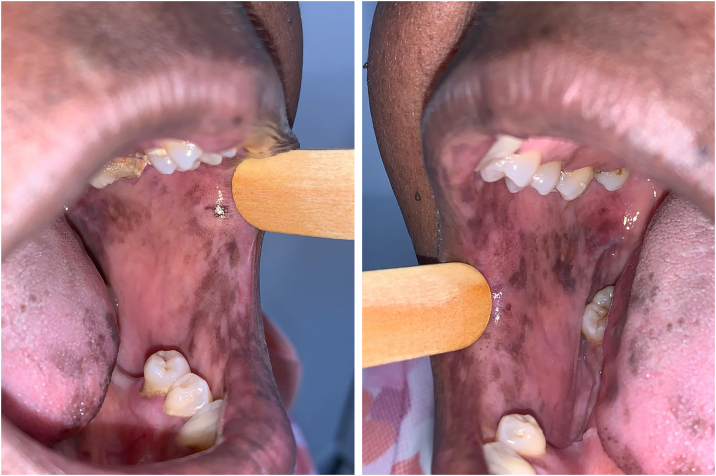
Clinical aspect of hyperpigmented spots on the bilateral buccal mucosa and tongue edges.

**Figure 4:**
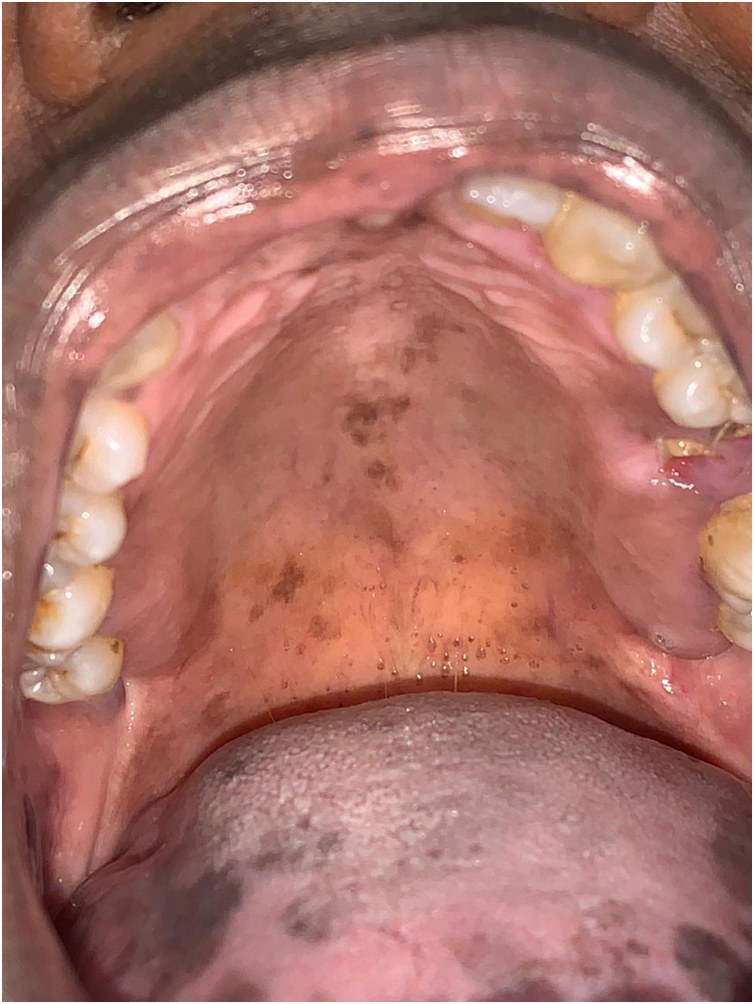
Hyperpigmented spots on the hard palate.

**Figure 5:**
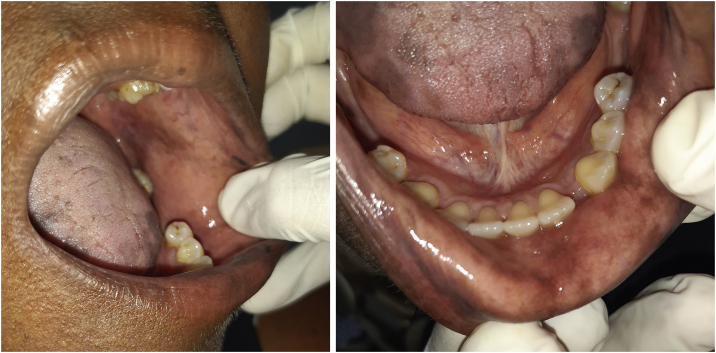
Attenuation of pigmented spots on the oral mucosa, when the medication was interrupted.

The patient was referred to the dentistry service at CEON/HUOC/UPE. On the extraoral examination, perioral hyperpigmentation and pigmented spots were observed on the hands and feet, affecting the dorsum and palms, especially in the region over the proximal interphalangeal and metacarpophalangeal joints, dorsum and instep. Moreover, hyperpigmented spots were identified intraorally, on the tongue and inner region of the lips (Fig. 2), on the jugal mucosa bilaterally (Fig. 3), and on the hard palate (Fig. 4); she was partially dentate, with a fractured tooth and unsatisfactory oral hygiene. The need for clinical restorative, periodontal dental treatment was detected and the Standard Operating Protocol for Oral Care (SOP-Oral) was instituted for the prevention and control of oral side effects from chemotherapy^15^. The patient was instructed about the contraindication of oral surgical procedures due to the use of zoledronic acid and its effects. During the follow-up consultations, a slight decrease in spots was observed on the oral mucosa (Fig. 5), hands and feet when the medication was withdrawn and an increase in hyperpigmentation when the cycle with capecitabine was restarted.

Currently, the patient is undergoing chemotherapy with capecitabine, mostly tolerating the regimen well, and the pigmented areas remain unchanged, with episodes of decrease and increase according to the chemotherapy cycle. She is being followed by the Oncology and Dentistry teams at CEON/HUOC/UPE.

## Discussion

The World Health Organization defines an adverse drug reaction as a response to a drug that is harmful and unintended, and that occurs at doses normally used in humans for the prophylaxis, diagnosis and treatment of diseases or for the modification of physiological functions^16^. In cancer patients, some of these reactions can be fatal, requiring greater caution by the healthcare provider^17^.

According to Sanches Junior et al.^10^, the identification of the adverse reaction pattern related to the triggering drug and the toxicity is extremely important for health professionals, as well as the differential diagnosis with infectious processes and specific manifestations of the neoplasm.

**Figure 6:**
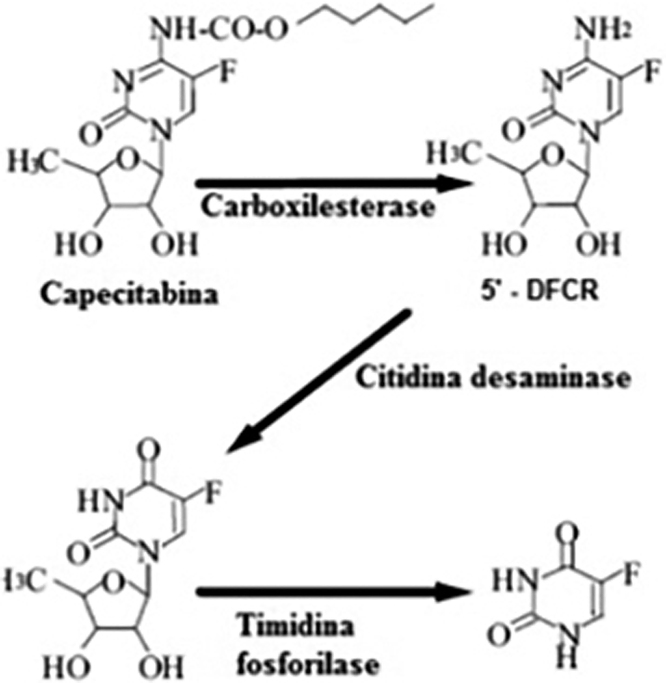
Metabolic pathway of the transformation of capecitabine into 5-Fluorouracil (5-FU). Source: Martins et al.^6^.

Capecitabine prodrug is easily absorbed from the gastrointestinal tract and is rapidly metabolized to 5-FU through a three-step enzymatic process (Fig. 6). In the first step, capecitabine is hydrolyzed by carboxylesterase in the liver to the intermediate 5′-deoxy-5-fluorocytidine (5′-DFCR). In the second step, 5′-DFCR is converted to 5′-Deoxy-5-Fluorouridine (5′-DFUR) by cytidine deaminase, which is highly active in the liver and tumor tissue. In the third step, 5′-DFUR is converted to 5-Fluorouracil (5-FU) by thymidine phosphorylase (TP), which is present in tumor tissue, resulting in the release of 5-FU preferentially in tumor tissue^5^, ^6^, ^7^.

Rodriguez et al.^18^ and Dean et al.^17^, report that once capecitabine is activated to 5-FU, it begins to exert its cytotoxic activity, inhibiting DNA synthesis, RNA processing, and protein synthesis, thereby causing cell damage and adverse effects.

According to Surjushe et al.^19^, the HFS, also known as palmar-plantar erythrodysesthesia or acral erythema, is an adverse reaction resulting from therapy with chemotherapeutic and biological agents and is mentioned by Rocha Filho^20^, Bispo Junior et al.^21^ and Costa et al.^22^ as the most common and limiting adverse effect of capecitabine use, diagnosed in approximately 50% of patients treated with this chemotherapeutical agent. The capecitabine package insert, based on post-sales information, indicates that the HFS was classified as a very common adverse reaction and, among all patients who developed the reaction, 17.0% had grade 3, as reported herein^23^.

The mechanism that leads to the occurrence of HFS is still unknown, but several theories have been proposed. A mechanism proposed by Cruz et al.^24^ and Simão et al.^25^ consists of the extravasation of antineoplastic agents through the capillaries, mainly of the hands and feet, as they are sites in the body that are more subject to trauma due to daily activities, leading to damage to local and underlying tissues, and also due to specific characteristics of the hands and feet, such as greater exposure to temperature variations, differentiated microvascularization, large amounts of keratinocytes, eccrine glands and epidermal cells undergoing constant cell division.

Milano et al.^5^, explain that the occurrence of HFS in patients who use capecitabine could also be justified by the presence of the TF enzyme, responsible for metabolizing the prodrug into 5-FU in keratinocytes, which could lead to the local production and accumulation of active metabolites in the hands and feet, damaging skin cells.

The diagnosis of HFS is based on its clinical characteristics and, in this case report, the patient was diagnosed with all degrees at different moments. When in the most debilitating stage, it was necessary to pause the medication, conduct recommended by Farr and Safwat^26^, who reported that the most effective management of HFS consists of increasing the interval between the administrations of capecitabine, decreasing the dose or interrupting the treatment until toxicity is reduced to grade 1 or zero. Other measures for the treatment of HFS include the use of topical emollients, antibiotics to prevent secondary infections, topical steroids, pyridoxine, and COX-2 inhibitors^27^.

Most case reports found in the literature are female patients, as can be seen in a study carried out by Costa et al.^22^, who reported that the female population predominated among cases of HFS, with 53.3% of women and 46.6% of men, and Miller et al.^28^ who considered the female sex as a risk factor for the development of HFS. Also in the study carried out by Costa et al.^22^, it was observed that the mean age of patients who developed HFS was 57 years, different from Rocha Filho^20^, who pointed out that he did not observe any predilection for age and gender for the presentation of HFS when using capecitabine.

Studies by Narasimhan et al.^29^ and Saif and Sandoval^30^ found a higher frequency of HFS among black patients treated with capecitabine than in white patients under the same therapeutic regimen and also observed that capecitabine administered at the recommended doses in black people leads to hyperpigmentation of the palms and soles, which has not yet been reported in white patients. Thus, the studies indicate that the pattern of manifestations of HFS is different between different ethnic groups, and corroborate the clinical characteristics found in the black patient of the present report, who also had hyperpigmentation on the hands and feet. Vasudevan^31^, states that hyperpigmentation of the hands and feet, rather than erythema, is considered the initial manifestation in most patients.

Saif and Sandoval^30^ even recommend that another classification be made, based on the characteristics found in HFS in black people:

• Grade 1: Hyperpigmentation of the palms and soles;
• Grade 2: Thickening of the skin on the palms and soles, with desquamation, pain and loss of function;
• Grade 3: Ulceration, dermatitis or moisty desquamation, severe pain and loss of function.

According to information from the drug package insert, hyperpigmentation occurred in less than 5% of patients included in the clinical studies conducted during the development of capecitabine^23^.

Hyperpigmentation is known to occur at the same time as HFS, although it is unclear whether the two manifestations are related or are separate conditions that occur simultaneously, however, most studies treat it as a variation and part of the initial presentation of HFS^32^.

Variations of HFS can be found, such as keratoderma, scleroderma, melanonychia, and skin pigmentations, but according to Vasudevan^31^, his study was the first to report and describe a case of oral pigmentation associated with HFS, as a side effect of capecitabine use. The patient in the case described by Vasudevan^31^, was a 59-year-old male black individual, who during therapy with capecitabine, in addition to showing hyperpigmentation of both feet and hands, had hyperpigmented spots on the dorsum of the tongue.

Subsequently, Caprez et al.^32^ seemingly described the 2nd case report in the literature of oral pigmentation associated with the use of capecitabine. This case described an 80-year-old black female patient, who after starting chemotherapy presented hyperpigmentation of the hands, feet and face, and hyperpigmented spots on the tongue and upper and lower alveolar ridge.

Both previously reported cases are similar to the present one regarding association with HFS and the sites showing spots on the oral mucosa, which are quite uncommon and rare.

Sweetman^9^ and Cury-Martins et al.^4^, mention skin pigmentation disorders and mucosal hyperpigmentation in their study as adverse events that may occur with the use of capecitabine, but the exact pathophysiology is yet to be well understood. According to Alfreijat^33^, it has been suggested that the melanocyte-stimulating hormone (MSH) may be potentiated by certain chemotherapeutic agents and this may be responsible for the higher prevalence of this adverse effect in dark-skinned patients. Neville et al.^34^, state that, as in many cases of increased melanin pigmentation, women are more sensitive, most likely because there is an interaction with sex hormones.

Other studies report cases of oral pigmentation involving other chemotherapeutic agents, such as the cases reported by Blaya and Saba^35^ and Alfreijat^33^, who report the appearance of hyperpigmented asymptomatic spots on the tongue of patients, without any involvement in other parts of the body, during chemotherapy with doxorubicin and cyclophosphamide. According to Neville et al.^34^, oral mucosal pigmentation related to chemotherapeutical drugs is most commonly associated with the use of doxorubicin, busulfan, cyclophosphamide, or 5-fluorouracil.

Several drugs have been considered to cause pigmentation on the buccal mucosa. These pigmentary changes have been associated with the use of phenolphthalein, minocycline, tranquilizers, antimalarials, estrogen, chemotherapeutic agents, and some antiretroviral drugs^34^. Neville et al.^34^ also mention that the clinical presentations of pigmentation related to drug use vary and any mucosal surface can be involved, but the attached gingiva and buccal mucosa are the most affected sites and may have a similar appearance to racial pigmentation.

In the present case, the patient had diffuse spots, with a blackish color and irregular edges, spread over the borders and dorsum of the tongue, bilateral buccal mucosa, hard palate, perioral and inner lip regions, without need for treatment, although there were aesthetic concerns. A significant improvement in HFS and hyperpigmentation was observed when the medication was discontinued, confirming that HFS with oral hyperpigmentation is associated with the use of the antineoplastic drug capecitabine.

## Conclusion

It is essential to identify and understand the side effects of antineoplastic drugs, considering the need to control them to favor the successful treatment and patient quality of life during and after antineoplastic therapy.

Oral hyperpigmentation induced by the use of capecitabine and associated with hand-foot syndrome is already a known reality, although further studies are still necessary on the addressed topic, aiming to clarify doubts and provide more explanations about its pathophysiology, in addition to new updates to the package insert and classification of adverse effects. A wide variety of lesions and conditions can result in abnormal pigmentation of the skin and oral mucosa; therefore, it is expected that this study can contribute to the identification and correct diagnosis of hyperpigmentation in the oral cavity, as well as the identification of adverse effects related to capecitabine use.

## Financial support

State Health Secretariat, Recife, PE, Brazil.

## Authors' contributions

Anna Danielly Almeida do Nascimento: Design and planning of the study; drafting and editing of the manuscript.

Débora Maria Porto: Intellectual participation in the propaedeutic and/or therapeutic conduct of the studied cases.

Aurora Karla de Lacerda Vidal: Effective participation in research orientation.

## Conflicts of interest

None declared.
